# Hand-foot-mouth disease and use of steroids, intravenous immunoglobulin, and traditional Chinese herbs in a tertiary hospital in Shantou, China

**DOI:** 10.1186/s12906-018-2259-9

**Published:** 2018-06-20

**Authors:** Dangui Zhang, Jieling Chen, William Ba-Thein

**Affiliations:** 1 Research Center of Translational Medicine, Second Affiliated Hospital of Shantou University Medical College, Shantou, Guangdong 515040 People’s Republic of China; 2 Shantou-Oxford Clinical Research Unit, Shantou University Medical College, Shantou, Guangdong 515040 People’s Republic of China; 3 Department of Microbiology and Immunology, Shantou University Medical College, 11/F, Science & Technology Building, 22 Xinling Road, Shantou, Guangdong 515041 People’s Republic of China

**Keywords:** Epidemiology, Infectious diseases, Therapeutics

## Abstract

**Background:**

In contrast to the guidelines of World Health Organization (WHO) and United States-Centers for Disease Control and prevention (US-CDC), the Chinese national guidelines recommend the use of steroids, intravenous immunoglobulin (IVIG), or traditional Chinese herbs (TCHs) in hand-foot-mouth disease (HFMD) management. Their use and therapeutic efficacies are, however, unclear. We aimed to describe their use in and the clinical outcomes of hospitalized HFMD cases.

**Methods:**

A retrospective review of hospital medical records for HFMD cases during 2008–2016 was conducted in a medical school-affiliated tertiary hospital in Shantou, Guangdong, China.

**Results:**

Hospitalized children with the discharge diagnosis of HFMD (*n* = 3778), comprising mild (58.4%), severe (41.5%), and very severe (0.1%) cases, were enrolled in the study. Steroids, IVIG, and antiviral TCH Lan-Qin were respectively prescribed in 60.5, 37.1, and 71.0% of cases. Most cases (99.8%) recovered and six died. Recovery rate was lower with the use of IVIG and higher with Lan-Qin (alone or in combination with steroid) in the mild cases (*Ps <* 0.05). Longer hospital stay was observed with steroid/IVIG with or without Lan-Qin in the severe cases (*Ps <* 0.05).

**Conclusions:**

This nine-year retrospective review shows 1) an increase in the incidence of HFMD as well as the use of steroids, IVIG, and TCH over time, 2) no significant advantage of using steroids and IVIG, either alone or in combination, in the management of mild HFMD cases, and 3) a higher recovery rate in mild HFMD cases with the use of antiviral TCH (Lan-Qin). Our findings need verification in a larger prospect study with cases from hospitals in other regions of China. Lan-Qin efficacy should be evaluated in randomized trials. Meanwhile, caution should be exercised in the extensive use of steroids and IVIG in HFMD management.

**Electronic supplementary material:**

The online version of this article (10.1186/s12906-018-2259-9) contains supplementary material, which is available to authorized users.

## Background

Hand-foot-mouth disease (HFMD) is a common viral illness that usually affects infants and children younger than 5 years old. Although large outbreaks of HFMD are not common in developed countries, outbreaks are large and occur often in the Asia-Pacific region [1].

Classic symptoms of HFMD include acute onset of fever with rashes in mouth, hands, feet, knees, and buttocks that usually resolve in 7–10 days. Some cases can become severe resulting in neurological and cardiopulmonary complications with high mortality [2]. The annual incidence rate in China was 1.2 per 1000 person-years during 2010–2012, with 0.03% case fatality [3].

Enterovirus 71 (EV 71) and Coxsackievirus A 16 (CA 16) are responsible for most HFMD cases. Although, the pathogenesis of HFMD is not fully understood, inflammatory mediators (cytokines and chemokines) are reportedly associated with HFMD severity [4]. It has also been shown that anti-IL-6 neutralizing antibody therapy in EV71-infected mice increases the survival rates and immune cell activation and reduces tissue damage, indicating the potential usefulness of immunoglobulins in HFMD management [5] .

Diagnosis of HFMD is primarily clinical. According to the World Health Organization (WHO) and United States-Centers for Disease Control and Prevention (US-CDC) guidelines, managing fever is the focus for mild cases; for severe cases, management is still largely supportive as there are no specific antivirals [2, 6]. Following the first outbreak of HFMD in 2008 in China, the Health Ministry of China published the guidelines for HFMD diagnosis and treatment, which is yearly updated [7–9]. However, in contrast to the WHO [2] or US-CDC guidelines [6], the Chinese national guidelines recommend the use of steroids and intravenous immunoglobulin (IVIG) for severe cases and traditional Chinese herbs (TCHs) for all HFMD cases [8]. Since the use and therapeutic efficacies of steroids, IVIG, and/or TCHs in HFMD remain unclear, we aimed to describe their use in hospitalized HFMD cases and the clinical outcomes.

## Methods

### Study design and setting

This study is a retrospective medical record review of HFMD cases in a medical school-affiliated hospital in Shantou, Guangdong, China. This 1500-bed hospital is one of the 3 tertiary referral hospitals in Shantou, serving a population of 5.3 million (as of 2010).

### Case definitions and selection criteria

Following the Health Ministry of China [8], HFMD (ICD-10-B08.4) cases were defined and classified clinically as: 1) mild: having papulovesicular rashes on the palms and soles, and multiple oral (mouth/tongue) ulcers, with or without fever; 2) severe: HFMD with central nervous system (CNS) involvement (lethargy, drowsiness, delirium, headache, vomiting, limb shaking, ataxia, nystagmus, oculomotor palsy, bulbar palsy, Acute Flaccid Paralysis, or convulsion); and 3) very severe: severe HFMD with cardiopulmonary involvement such as (i) seizures, coma, or cerebral hernia, (ii) respiratory distress, cyanosis, bloody frothy sputum, or lung rales, or (iii) poor peripheral perfusion such as shock.

All hospitalized children with the discharge diagnosis of HFMD during 2008–2016 were included and cases with incomplete therapeutic information were excluded from this study.

### Data collection and analysis

Patients’ medical records were reviewed for demographic data, clinical features, management, and outcomes. SPSS version13 (SPSS, Chicago, IL) was used for statistical analysis. Association between categorical variables was analyzed by Chi-square test, normally distributed continuous variables by T-test, and non-normally distributed data by Mann-Whitney U test. All statistical tests were two-tailed, and *p*-value **<** 0.05 was considered statistically significant.

## Results

### Characteristics of HFMD cases (Table 1)

In total, there were 3778 HFMD cases, including 58.4% of mild, 41.5% of severe, and 0.1% of very severe cases, during nine-year study period. More than 90% of cases were **<** 4 years old and around 60% were **<** 2 years old; whereas 41.9% of severe and 75.0% of very severe cases were 1–2 years old. Male-female ratio was 1.6. There was no significant association between age or gender and HFMD severity.

**Table 1:** The characteristics, intervention, and outcome of HFMD cases (2008–2016) ^$^

		Total	Mild cases	Severe cases	Very severe cases
		*N* = 3778	*n* = 2208	*n* = 1566	*n* = 4
Gender	Male	2351 (62.2)	1357 (61.5)	992 (63.3)	2 (50.0)
	Female	1427 (37.8)	851 (38.5)	574 (36.7)	2 (50.0)
Age (year)	< 1	890 (23.6)	582 (26.4)	308 (19.7)	0 (0)
	1 - < 2	1471 (38.9)	812 (36.8)	656 (41.9)	3 (75.0)
	2 - < 3	832 (22.0)	468 (21.2)	364 (23.2)	0 (0)
	3 - < 4	372 (9.9)	209 (9.4)	162 (10.3)	1 (25.0)
	4–11	213 (5.6)	137 (6.2)	76 (4.9)	0 (0)
Intervention					
No steroid/IVIG/Lan-Qin		415 (11.0)	368 (16.7)	47 (3.0)	0 (0)
Steroid only		278 (7.4)	194 (8.8)	84 (5.4)	0 (0)
IVIG only		43 (1.1)	22 (1.0)	21 (1.3)	0 (0)
Lan-Qin only		946 (25.0)	840 (38.0)	106 (6.8)	0 (0)
Steroid + IVIG		359 (9.5)	92 (4.2)	264 (16.9)	3 (75.0)
Steroid + Lan-Qin		738 (19.5)	521 (23.6)	217 (13.9)	0 (0)
IVIG + Lan-Qin		88 (2.3)	41 (1.9)	47 (3.0)	0 (0)
Steroid + IVIG + Lan-Qin		911 (24.1)	130 (5.9)	780 (49.8)	1 (25.0)
Outcome					
Recovery		3470 (91.8)	2082 (94.3)	1387 (88.6)	0 (0)
Death		6 (0.2)	0 (0)	2 (0.2)	4 (100)
Self-discharge		302 (8.0)	126 (5.7)	176 (11.2)	0 (0)
Length of hospital stay for all cases		4.70 ± 2.6	4.09 ± 1.8	5.40 ± 3.0	14.25 ± 12.0
Length of hospital stay without self-discharged cases		4.69 ± 2.5	4.13 ± 1.7	5.49 ± 3.2	14.25 ± 12.0

^$^data are shown as n (%). Lan-Qin was the most prescribed drug among 8 traditional Chinese herbs (TCH)

### Epidemiology (Fig. 1)

The monthly incidence rate of HFMD cases increased sharply from 13 in 2008 to a maximum of 720 in 2012, with a monthly mean of 35 cases and an annual mean of 420 cases. The monthly and annual incidence rates of severe cases surpassed that of mild cases in 2014. There existed a pattern of semiannual outbreaks during the study period: one in early summer (May) and the other in mid-autumn (September to October), except for 2013 and 2015.

**Fig. 1:**
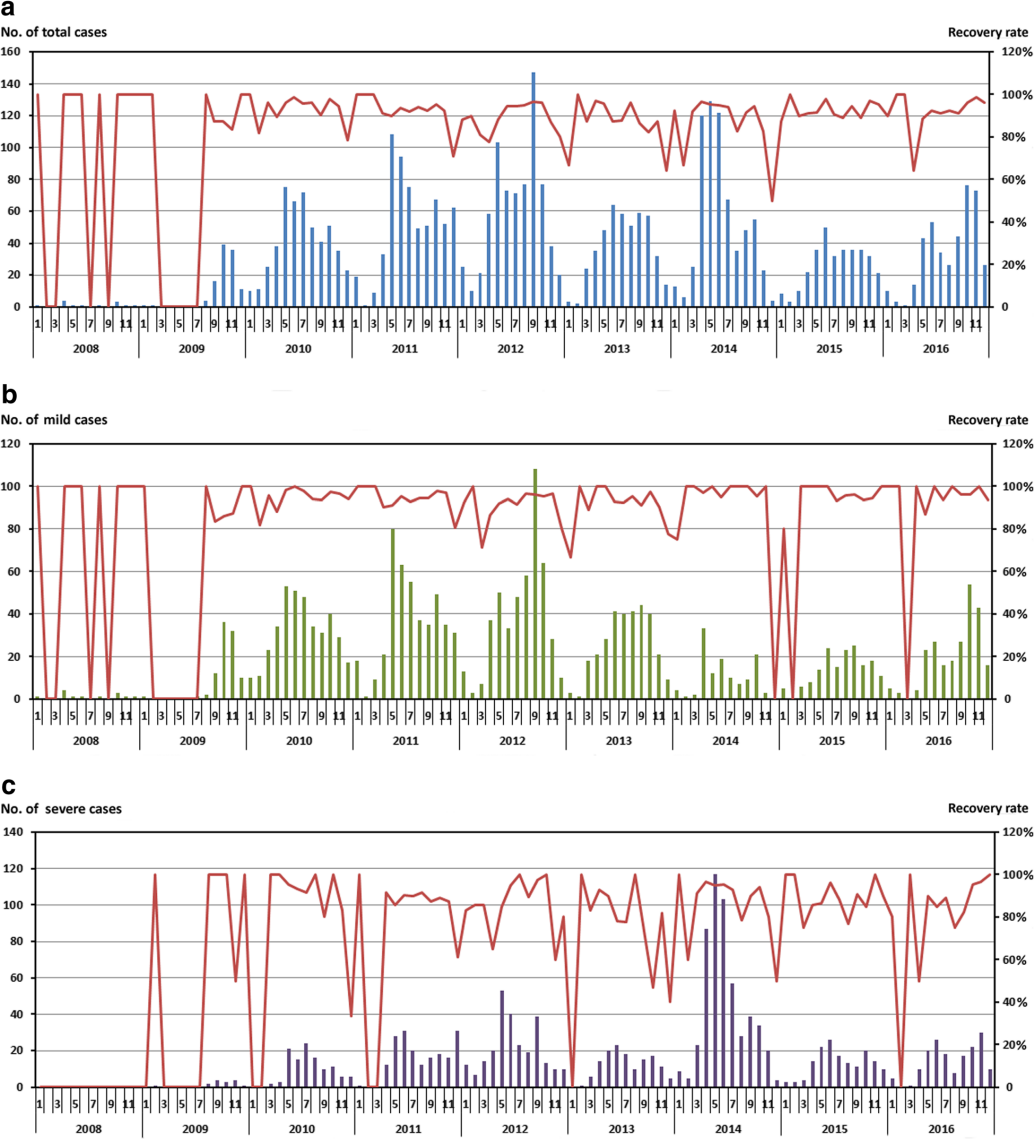
Monthly incidence and recovery rates of HFMD during 2008–2016. Recovery is defined as clearance of fever, rashes, and CNS and cardiopulmonary complications

### Interventions (Table 1)

All HFMD cases in the study received conventional and supportive therapy, such as antipyretics and fluid infusion. Steroids, IVIG, and TCHs were additionally prescribed in 89.0% of patients (3363/3778). Three types of steroid—methyl-prednisone, prednisolone, or dexamethasone—were prescribed in 60.5% (2286/3778) of total cases, or 42.4% (937/2208) of mild cases, 85.9% (1345/1566) of severe cases, and 100% (4/4) of very severe cases. Annual prescription rate of steroids went up from 23.1% in 2008 to 88.3% in 2014, with approximately 50% in other years (see Additional file 1).

IVIG was prescribed in 37.1% (1401/3778) of cases in total, or 13.0% (286/2208) of mild cases, 71.0% (1112/1566) of severe cases, and 100% (4/4) of very severe cases. Annual prescription rate of IVIG was approximately 30% in most study years, with the highest rate (74%) in 2014.

Eight TCHs were prescribed in 82.0% of patients (3097/3778) as antiviral (viz., Lan-Qin, Shuang-Huang-Lian, Si-Ji, Qing-Kai-Ling), cough suppressant (viz., Fei-Li-Ke, Xiao-Er-Zhi-Ke), antacid (viz., Si-Mo-Tang), and mucolytic (viz., Tan-Re-Qing) (data not shown). Antiviral Lan-Qin was predominantly prescribed alone or in combination with steroids and/or IVIG in 71.0% of cases, or exclusively in 840 mild and 106 severe cases accounting for 25% of cases. Lan-Qin use escalated significantly from 7.7% in 2008 to 81.4% in 2016 (*P* **<** 0.05).

### Outcomes

**Fig. 2:**
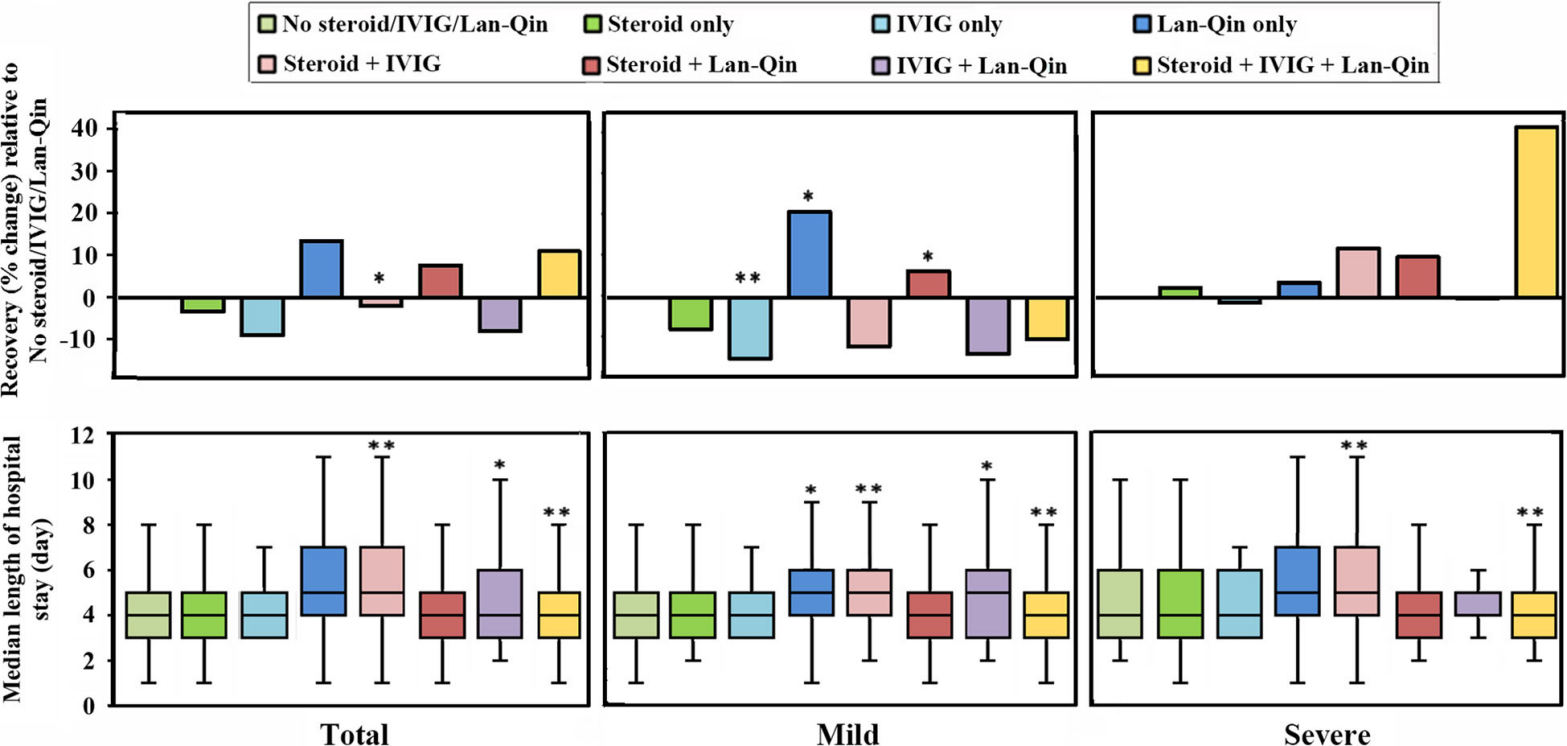
The outcomes as relative recovery rates (upper panel) and median length of hospital stay (lower panel) of HFMD cases treated with steroids, IVIG, and Lan-Qin (2008–2016); * *P* < 0.05, ** *P* < 0.01 as compared to no steroid/IVIG/Lan-Qin group

Most cases (99.8%) recovered and six died—two from the severe group (0.1%, 2/1566) and four from the very severe group (100%, 4/4). The causes of death for both severe cases were CNS complications and upper GI bleedings. Four deaths in very severe group were due to cardiopulmonary failure. The recovery rate of the mild cases was lower with the use of IVIG only (*P* < 0.01) and higher with the use of Lan-Qin only or steroid/Lan-Qin (*Ps* < 0.05), compared to no steroid/IVIG/Lan-Qin (Fig. 2). In total, however, steroid/IVIG use was associated with lower recovery (*P* < 0.005).

Longer hospital stay was observed with the use of steroid/IVIG, IVIG/Lan-Qin, or steroid/IVIG/Lan-Qin in the mild cases (*Ps* **<** 0.05~ 0.01), and steroid/IVIG or steroid/IVIG/Lan-Qin in the severe cases (*Ps* < 0.01) (Fig. 2). Overall, hospital stay was longer with the use of steroid/IVIG, IVIG/Lan-Qin, and steroid/IVIG/Lan-Qin (*Ps* **<** 0.05~ 0.01). A significant increase in the recovery rate and reduction in the duration of hospitalization was observed with solitary use of Lan-Qin in the mild cases only (Table 2). There were 302 (8.9%) self-discharged cases with unknown outcomes (see Additional file 2).

**Table 2:** Lan-Qin effect on the outcome of HFMD cases ^$^

	Total	Recovery^&^	Death	Self-discharge	Length of hospital stay (day)	
					All cases	Without self-discharged cases
Mild cases	1900	1807	0	93	4.09 ± 1.8	4.09 ± 1.7
No Lan-Qin/Other therapies^#^	368 (19.4)	348 (19.3)	0 (0)	20 (21.5)	4.03 ± 1.6	4.07 ± 1.5
Lan-Qin only	840 (44.2)	811 (44.9) *	0 (0)	29 (31.2)	3.83 ± 1.5 *	3.89 ± 1.4 *
Lan-Qin + Other therapies	692 (36.4)	648 (35.9)	0 (0)	44 (47.3)	4.43 ± 2.1	4.40 ± 2.0 **
Severe cases	1196	1061	2	133	5.40 ± 3.0	5.47 ± 2.9
No Lan-Qin/Other therapies	47 (3.9)	41 (3.9)	0 (0)	6 (4.5)	4.32 ± 2.1	4.39 ± 2.2
Lan-Qin only	106 (8.9)	97 (9.1)	0 (0)	9 (6.8)	4.25 ± 2.0	4.32 ± 2.0
Lan-Qin + Other therapies	1043 (87.2)	923 (87.0)	2 (100)	118 (88.7)	5.57 ± 3.1 **	5.64 ± 3.0 **
Very severe cases	1	0	1	0	16	16
No Lan-Qin/Other therapies	0 (0)	0 (0)	0 (0)	0 (0)	–	–
Lan-Qin only	0 (0)	0 (0)	0 (0)	0 (0)	–	–
Lan-Qin + Other therapies	1 (100)	0 (0)	1 (100)	0 (0)	16	16
Total	3097	2868	3	226	4.60 ± 2.4	4.60 ± 2.3
No Lan-Qin/Other therapies	415 (13.4)	389 (13.6)	0 (0)	26 (11.5)	4.07 ± 1.6	4.11 ± 1.6
Lan-Qin only	946 (30.5)	908 (31.7)	0 (0)	38 (16.8)	3.88 ± 1.6 *	3.89 ± 1.5
Lan-Qin + Other therapies	1736 (56.1)	1571 (54.8)	3 (100)	162 (71.7)	5.12 ± 2.8 **	5.14 ± 2.7 **

^$^Self-discharged cases (*n* = 302), and the cases treated with steroid only, IVIG only, and steroid/IVIG (*n* = 602) were excluded from the analysis. ^&^Recovery is defined as clearance of fever, erythema, and CNS and cardiopulmonary complications. ^#^Lan-Qin was the predominant drug among 8 TCHs prescribed; other therapies include steroids, IVIG, or combination of steroids and IVIG

Data are shown as n (%), or mean ± SD for length of hospital stay. *P < 0.05, **P < 0.01, vs. No Lan-Qin/Other therapies (by **χ**^**2**^ test for recovery and t-test for length of hospital stay)

## Discussion

This study presents the HFMD epidemiology and prescribing practices of steroid, IVIG, and TCHs in management of HFMD over nearly a decade in a Chinese tertiary referral hospital in Shantou.

We observed the increasing incidence rates of HFMD, a semiannual outbreak pattern (except in 2013 and 2015), the **<** 2 age group being most susceptible, and higher incidence in males during 2008–2016, which is consistent with the China CDC’s reports [3]. Since no gender preference for HFMD has been reported previously, higher male proportion in our study could be due to biased parental health-seeking behavior for boys in the Chaoshan region [10], where the study was done.

Self-limiting mild cases, which accounted for more than half of the hospitalized cases, were likely admitted to the hospital out of parental anxiety, especially when there were severe cases and deaths during the outbreaks. This is quite concerning because 83% of the mild cases received steroid, IVIG, and/or TCHs. Although steroid is not recommended for HFMD regardless of the severity in the WHO [2, 6] and CDC guidelines [2, 6], it is recommended in the Chinese national guidelines exclusively for severe HFMD cases with CNS or cardiopulmonary complications [8]. IVIG is mentioned in the WHO guidelines for HFMD management [2]; nevertheless, the recommendation is weak as it was based only on retrospective observational studies [11–13]. China CDC also suggests IVIG for severe HFMD cases in the 2008 national guidelines [8]. Using early and enough methylprednisone in combination with IVIG has been reported to have a positive effect (clearance of fever, erythema, and neurological symptoms) in severe cases in China [14]. The observed extensive use of steroids and IVIG in the mild cases could be due to physician’s prescribing behavior following the Expert Consensus on the management of severe EV71 HFMD cases (2011 version, the health ministry of China) [15], where steroids and IVIG are recommended for mild HFMD cases with a high risk of developing into severe cases. High-risk HFMD mild cases are defined as having one of the five predictors: continuously high fever (Temp. > 39 °C), CNS symptoms (lethargy, fatigue, vomiting, seizures), breathing abnormalities (tachypnea or bradypnea), circulatory disturbances (HR > 140–150/min or capillary filling time > 2 s), WBC > 15X10^9^/L, and blood glucose> 8.3 mmol/L. High-risk cases were, however, not identified in our review, suggesting an inappropriate prescribing behavior.

In Traditional Chinese Medicine, HFMD is classified as an infectious disease caused by dampness and heat with accumulation of heat into spleen, leading to symptoms appearing in mouth or on hands and feet [16]. Many TCHs are therefore recommended in the Chinese national guidelines for HFMD treatment, but their uses vary considerably in different hospitals in China [17]. Also, the recommendations are neither supported nor based on the high quality trials [18]. One systematic review of randomized clinical trials in China has reported that TCHs may improve HFMD symptoms [18].

Lan-Qin, the main TCH in this study, was predominantly prescribed, with likely reasons as cheap price (3 RMB or 0.45 US$ for 10 ml/dose), oral formulation, and potential therapeutic benefits. In fact, Lan-Qin significantly shortened the recovery rate of the mild HFMD cases when given alone or in combination with steroid although it showed no significant effect on severe cases. The efficacies of other TCHs for HFMD have been validated in both basic science and clinical studies [19–22]. Two commonly used herbs—*Houttuynia cordata* Thunb and Mentha haplocalyx Briq—recommended for HFMD by the China national guidelines have been shown to have significant anti-viral and anti-inflammatory activities: *Houttuynia cordata* Thunb inhibited EV-71 replication and Mentha haplocalyx Briq suppressed CA16 infection [21]. One double-blind comparative study showed that, in combination with the conventional therapy, TCH Jinlianqingre (tablet) shortened the time of both fever clearance and healing in the mild HFMD cases [19]. Another RCT study showed that TCH Jinzhen (oral liquid) significantly reduced the time of rash disappearance and fever clearance [22]. However, as described previously [20], the untoward effects of TCHs, especially when used in combination with other drugs in HFMD management—the information not available from retrospective medical record review—should be investigated prospectively in future research.

Our outcome analysis showed no significant advantage for the mild cases treated with steroid and IVIG, either alone or in combination; even a lower recovery rate or longer hospital stay was observed in this study. Given that both steroids and IVIG are not without potential adverse effects [2], prescribing them in a considerably high proportion of HFMD cases, especially the mild ones, should be cautious.

### Study limitations

Being a retrospective design, this study is not without potential biases: i) high-risk HFMD mild cases were neither documented in the medical records nor identified by our review, disenabling us to do independent outcome analysis of this group; ii) the outcome of the self-discharged patients was not known as follow up information was not available; iii) assessment of outcomes (such as the time of fever clearance or rash disappearance and the rate of severe HFMD development) with the use of steroid, IVIG, or TCHs was not possible due to lack of data in the medical records.

## Conclusions

This nine-year study shows 1) an increase in the incidence of HFMD as well as the use of steroids, IVIG, and TCHs over time in the study hospital, 2) no significant advantage of using steroids and IVIG, either alone or in combination, in the management of mild HFMD cases, and 3) a higher recovery rate in mild HFMD cases with the use of antiviral TCH (Lan-Qin). Our findings need verification in a larger prospect study with cases from hospitals in other regions of China. Lan-Qin efficacy should be evaluated in randomized trials. Meanwhile, caution should be exercised in the extensive use of steroids and IVIG in HFMD management.

## Additional files

Additional file 1: **Table S1**. Steroid, IVIG, and Lan-Qin use in HFMD cases by year (2008–2016) (DOCX 18 kb)

Additional file 2: **Table S2**. The interventions and outcomes of HFMD cases (2008–2016) (DOCX 23 kb)

## Abbreviations

CDC: Centers for Disease Control and Prevention
CNS: Central Nervous System
HFMD: Hand-foot-mouth disease
IVIG: Intravenous immunoglobulin
*Ps*: *P* values
TCHs: Traditional Chinese herbs
WHO: World Health Organization
