# Supplementary material for: Hand-foot-mouth disease and use of steroids, intravenous immunoglobulin, and traditional Chinese herbs in a tertiary hospital in Shantou, China
Source: BMC Complement Altern Med. 2018 Jun 20;18:190. doi: 10.1186/s12906-018-2259-9 (PMC6011388; doi:10.1186/s12906-018-2259-9)
Supplement: Supplementary file 1 — Table S1. Steroid, IVIG, and Lan-Qin use in HFMD cases by year (2008–2016) (DOCX 18 kb) [file 12906_2018_2259_MOESM1_ESM.docx]

Additional file 1 **Table S1** Steroid, IVIG, and Lan-Qin use in HFMD cases by year (2008-2016)

|  | **Total** | **Year** | | | | | | | | |
| --- | --- | --- | --- | --- | --- | --- | --- | --- | --- | --- |
|  |  | 2008 | 2009 | 2010 | 2011 | 2012 | 2013 | 2014 | 2015 | 2016 |
| Steroid only | 278 (7.4) | 2 (15.4) | 8 (7.3) | 48 (9.7) | 27 (4.4) | **55 (7.6)** | 40 (8.9) | 48 (7.4) | 21 (6.5) | 29 (7.2) |
| IVIG only | 43 (1.1) | 0 (0) | 0 (0) | 2 (0.4) | 4 (0.6) | 16 (2.2) | **17 (3.8)** | 1 (0.2) | 2 (0.6) | 1 (0.2) |
| Lan-Qin only | 946 (25.0) | 7 (53.8) | 52 (47.7) | 125 (25.2) | 68 (11.0) | 203 (28.2) | 69 (15.4) | **218 (33.7)** | 84 (26.1) | 120 (29.8) |
| Steroid + IVIG | 359 (9.5) | 1 (7.7) | 1 (0.9) | 53 (10.7) | 82 (13.2) | 52 (7.2) | **84 (18.8)** | 39 (6.0) | 20 (6.2) | 27 (6.7) |
| Steroid + Lan-Qin | 738 (19.5) | 1 (7.7) | 24 (22.0) | 123 (24.7) | 84 (13.5) | 131 (18.2) | 47 (10.5) | **159 (24.6)** | 74 (23.0) | 95 (23.6) |
| IVIG + Lan-Qin | 88 (2.3) | 0 (0) | 1 (0.9) | 22 (4.4) | 12 (1.9) | **28 (3.9)** | 5 (1.1) | 7 (1.1) | 1 (0.3) | 12 (3.0) |
| Steroid + IVIG + Lan-Qin | 911 (24.1) | 2 (15.4) | 10 (9.2) | 85 (17.1) | **322 (51.9)** | 129 (17.9) | 55 (12.3) | 108 (16.7) | 98 (30.4) | 102 (25.3) |
| Steroid (total) | 2286 (60.5) | 3 (23.1) | 57 (52.3) | 247 (49.7) | 370 (59.7) | 426 (59.2) | 261 (58.6) | **573 (88.3)** | 184 (57.3) | 165 (40.8) |
| IVIG (total) | 1401 (37.1) | 3 (23.1) | 57 (52.3) | 154 (31.0) | 144 (23.3) | 237 (32.9) | 113 (25.4) | **483 (74.4)** | 95 (29.6) | 115 (28.5) |
| Lan-Qin (total) | 2683 (71.0) | 10 (76.9) | 87 (79.8) | 355 (71.4) | 486 (78.4) | 491 (68.2) | 176 (39.6) | **492 (75.8)** | 257 (80.1) | 329 (81.4) |
| No steroid/IVIG/Lan-Qin | 415 (11.0) | 1 (7.7) | 13 (11.9) | 39 (7.8) | 21 (3.4) | 106 (14.7) | **128 (28.8)** | 69 (10.6) | 21 (6.5) | 18 (4.5) |
| Total cases | 3778 (100) | 13 (100) | 109 (100) | 497 (100) | 620 (100) | 720 (100) | 445 (100) | 649 (100) | 321 (100) | 404 (100) |
