# Supplementary material for: Hand-foot-mouth disease and use of steroids, intravenous immunoglobulin, and traditional Chinese herbs in a tertiary hospital in Shantou, China
Source: BMC Complement Altern Med. 2018 Jun 20;18:190. doi: 10.1186/s12906-018-2259-9 (PMC6011388; doi:10.1186/s12906-018-2259-9)
Supplement: Supplementary file 2 — Table S2. The interventions and outcomes of HFMD cases (2008–2016) (DOCX 23 kb) [file 12906_2018_2259_MOESM2_ESM.docx]

Additional file 2 **Table S2.** The interventions and outcomes of HFMD cases (2008-2016)

|  | **Total** | **Recovery** | **Death** | **Self-discharge** | **Length of hospital stay (day)** | |
| --- | --- | --- | --- | --- | --- | --- |
|  |  |  |  |  | **All cases** | **Without self-discharged cases** |
| **Mild cases** | **2208** | **2082** | **0** | **126** | **4.09 ± 1.8** | **4.13 ± 1.7** |
| No steroid/ IVIG/ Lan-Qin | 368 (16.7) | 348 (16.7) | 0 (0) | 20 (15.9) | 4.03 ± 1.6 | 4.07 ± 1.5 |
| Steroid only | 194 (8.8) | 176 (8.5) | 0 (0) | 18 (14.3) | 4.02 ± 1.4 | 4.05 ± 1.3 |
| IVIG only | 22 (1.0) | 17 (0.8) ** | 0 (0) | 5 (4.0) | 4.23 ± 1.3 | 4.35 ± 1.2 |
| Lan-Qin only | 840 (38.0) | 811 (39.0) * | 0 (0) | 29 (23.0) | 3.83 ± 1.5 | 3.89 ± 1.4 * |
| Steroid + IVIG | 92 (4.2) | 82 (3.9) | 0 (0) | 10 (7.9) | 5.53 ± 2.5 ** | 5.53 ± 2.1 ** |
| Steroid + Lan-Qin | 521 (23.6) | 488 (23.4) * | 0 (0) | 33 (26.2) | 4.19 ± 1.8 | 4.16 ± 1.7 |
| IVIG + Lan-Qin | 41 (1.8) | 40 (1.9) | 0 (0) | 1 (0.8) | 4.90 ± 2.3 * | 5.00 ± 2.2 ** |
| Steroid + IVIG + Lan-Qin | 130 (5.9) | 120 (5.8) | 0 (0) | 10 (7.9) | 5.25 ±2.6 ** | 5.18 ±2.6 ** |
| **Severe cases** | **1565** | **1387** | **2** | **176** | **5.40 ± 3.0** | **5.49 ± 3.2** |
| No steroid/ IVIG/ Lan-Qin | 47 (3.0) | 41 (3.0) | 0 (0) | 6 (3.4) | 4.32 ± 2.1 | 4.39 ± 2.2 |
| Steroid only | 84 (5.4) | 78 (5.6) | 0 (0) | 6 (3.4) | 4.89 ± 3.2 | 5.01 ± 3.3 |
| IVIG only | 21 (1.3) | 20 (1.4) | 0 (0) | 1 (0.6) | 5.10 ± 2.6 | 5.10 ± 2.7 |
| Lan-Qin only | 106 (6.8) | 97 (7.0) | 0 (0) | 9 (5.1) | 4.25 ± 2.0 | 4.32 ± 2.0 |
| Steroid + IVIG | 264 (16.8) | 228 (16.4) | 0 (0) | 36 (20.5) | 5.93 ± 4.3 ** | 5.77 ± 4.0 ** |
| Steroid + Lan-Qin | 217 (13.9) | 194 (13.9) | 0 (0) | 23 (13.1) | 4.45 ± 2.5 | 4.52 ± 2.3 |
| IVIG + Lan-Qin | 47 (3.0) | 37 (2.7) | 0 (0) | 10 (5.7) | 4.47 ± 2.0 | 4.68 ± 2.1 |
| Steroid + IVIG + Lan-Qin | 779 (49.8) | 692 (49.9) | 2 (100) | 85 (48.3) | 5.94 ±3.2 ** | 6.00 ±3.2 ** |
| **Very severe cases** | **4** | **0** | **4** | **0** | **14.25 ± 12.0** | **14.25 ± 12.0** |
| Steroid + IVIG | 3 (75.0) | 0 (0) | 3 (75.0) | 0 (0) | 13.67 ± 14.6 | 13.67 ± 14.6 |
| Steroid + IVIG + Lan-Qin | 1 (25.0) | 0 (0) | 1 (25.0) | 0 (0) | 16 | 16 |
| **Total cases** | **3778** | **3470** | **6** | **302** | **4.70 ± 2.6** | **4.69 ± 2.5** |
| No steroid/ IVIG/ Lan-Qin | 415 (11.0) | 389 (11.2) | 0 (0) | 26 (8.6) | 4.07 ± 1.6 | 4.11 ± 1.6 |
| Steroid only | 278 (7.4) | 254 (7.3) | 0 (0) | 24 (7.9) | 4.28 ± 2.1 | 4.35 ± 2.2 |
| IVIG only | 43 (1.1) | 37 (1.1) | 0 (0) | 6 (2.0) | 4.7 ± 2.1 | 4.76 ± 2.2 |
| Lan-Qin only | 946 (25.0) | 908 (26.2) | 0 (0) | 38 (12.6) | 3.88 ± 1.6 | 3.89 ± 1.5 |
| Steroid + IVIG | 359 (9.5) | 311 (9.0) | 2 (33.3) | 46 (15.2) | 5.89 ±4.1 ** | 5.74 ± 3.8 ** |
| Steroid + Lan-Qin | 738 (19.5) | 682 (19.7) | 0 (0) | 56 (18.5) | 4.27 ± 2.0 | 4.26 ± 1.9 |
| IVIG + Lan-Qin | 88 (2.3) | 77 (2.2) | 0 (0) | 11 (3.6) | 4.67 ± 2.1 * | 4.84 ± 2.1 * |
| Steroid + IVIG + Lan-Qin | 911 (24.1) | 812 (23.4) * | 4 (66.7) | 95 (31.5) | 5.86 ±3.2 ** | 5.90 ± 3.1 ** |

Note: Recovery was defined as clearance of fever, erythema, and CNS and cardiopulmonary complications; steroids include ethyl-prednisone, prednisolone, and dexamethasone; Lan-Qin was the most prescribed drug among 8 traditional Chinese herbs (TCHs) in this study.

Data are shown as n (%) or mean ± SD for length of hospital stay. *P<0.05, **P<0.01, No steroid/IVIG/Lan-Qin vs. other groups (by **^ ^**test for recovery and t-test for the length of hospital stay).
